# Prevalence of suicidal ideation and suicide attempts among Iranian university students: systematic review and meta-analysis

**DOI:** 10.1192/bjo.2025.10921

**Published:** 2026-01-06

**Authors:** Seyyed Muhammad Mahdi Mahdavinoor, Aghil Mollaei, Nazanin Mousavi, Amir-Hassan Bordbari, Raziye Dehbozorgi, Leila Seddigh

**Affiliations:** Faculty of Allied Medical Sciences, Mazandaran University of Medical Sciences, Sari, Iran; Student Research Committee, Faculty of Health, Mazandaran University of Medical Sciences, Sari, Iran; Department of Social Science, Faculty of Psychology, Imam Khomeini International University, Qazvin, Iran; Student Research Committee, Faculty of Medicine, Mazandaran University of Medical Sciences, Sari, Iran; Namazi Hospital, Shiraz University of Medical Sciences, Shiraz, Iran; Department of Community Medicine, Tehran University of Medical Sciences, Tehran, Iran

**Keywords:** Suicide, students, epidemiology, meta-analysis, Iran

## Abstract

**Background:**

Students, due to their specific academic and psychosocial conditions, are at higher risk of suicide compared with the general population, and suicide is one of the leading causes of death among students worldwide.

**Aims:**

To investigate the prevalence of suicidal ideation and suicide attempts among Iranian university students.

**Method:**

A systematic search was conducted in international and national databases, including Scopus, Web of Science, PsycINFO, PubMed and Magiran, up to February 2025. Title and abstract screening was performed by a single reviewer. Two reviewers independently undertook full-text screening (study selection) and data extraction. Data were analysed using Stata 16. The heterogeneity of studies was tested with Cochran’s *Q* and quantified with the *I* ^2^ statistic. To explore the sources of heterogeneity, we performed sensitivity analyses and meta-regression. The protocol was registered in the International Registration of Systematic Reviews (no. CRD42023471340).

**Results:**

We included 28 studies in this research. The pooled prevalence of suicidal ideation, 12-month suicide attempts and lifetime suicide attempts among Iranian students was 17% (95% CI: 13–21%), 3% (95% CI: 2–4%) and 8% (95% CI: 6–10%), respectively, with substantial heterogeneity (*I* ^2^ = 94.85, 91.16 and 93.46%, respectively).

**Conclusions:**

This study highlights the high prevalence of suicidal ideation and suicide attempts among Iranian university students, underscoring the need for effective preventive strategies and further research.

The mental health status of university students has been discussed over many years.^1–6^ Admission to university brings about changes in individual, family and social life and is therefore regarded as a highly sensitive phase.^1^ The pressures imposed on students and their difficult circumstances make their mental health more vulnerable than that of the general population.^7,8^ This increased vulnerability to mental disorders among students can lead to a wide range of adverse outcomes, including suicide. In fact, students are at greater risk of suicidal than the non-student population,^9^ so that approximately one of every four students worldwide has experienced suicidal ideation in some way.^10^ Suicide is also a major public health concern in low- and middle-income countries: according to a World Health Organization report, 77% of all suicides occur in these countries.^11^ Poor screening, absence of comprehensive suicide prevention systems, lack of government investment in people’s mental health, class differences, poverty, social crises and so forth are probably among the contributory factors.^4^ The key issue about suicide is its effect on society. It has been reported that every successful suicide puts 135 more people at risk of suicide.^12^ This means that suicide does not increase in an arithmetical progression, but rather in a geometric progression. Despite the fact that suicide occurs more frequently in low- and middle-income countries, accurate information concerning its prevalence and associated factors is usually not available depending to the conditions of those countries. For example, there are limited and contradictory data with respect to the frequency of suicide in Iran.^13^ This lack of information complicates practical planning for the prevention of suicide.

In Iran, several studies have measured the prevalence of suicidal ideation and attempts among university students. Niusheh Soofi et al estimated the prevalence of suicidal ideation at 6% in students from Rafsanjan University of Medical Sciences.^14^ In another study at Tehran University of Medical Sciences, Nakhostin-Ansari et al approximated the prevalence of suicidal ideation among students to be 32%.^15^ Rohani and Esmaeili also found that 20% of students at the University of Isfahan had experienced suicidal ideation.^16^ Differing prevalence rates of suicide attempts have also been reported among Iranian students. For example, Poorolajal et al^17^ estimated the prevalence of suicide attempts among students at 13 universities of medical sciences in Iran to be 1.7%. In addition, another study at Ardabil University of Medical Sciences found that this rate was 7.9%.^18^

Student suicide in low- and middle-income countries appears to be a highly important matter given its high prevalence in these countries, in addition to the effect of each person’s suicide on other people and the high costs imposed on society. Despite the importance of suicide among students, to our knowledge there has been no systematic review of studies reporting the prevalence of suicidal ideation and suicide attempts among Iranian university students. Therefore, our goal was to conduct a systematic review and meta-analysis focusing on the prevalence of suicidal ideation and suicide attempts among this group. Our research can provide a comprehensive view of suicide among Iranian students.

## Method

### Study design

We developed and conducted this systematic review and meta-analysis in line with two methodological guides: the Cochrane Handbook for Systematic Reviews of Interventions^19^ and a step-by-step guide for conducting systematic reviews and meta-analyses with simulation data.^20^ We also reported this review according to the Preferred Reporting Items for Systematic Reviews and Meta-Analyses guideline.^21^ The protocol of this research has been registered with the International Registration of Systematic Reviews (PROSPERO) under the following registration number: CRD42023471340. Our review question was developed based on the CoCoPop^22^ framework, where ‘Condition’ refers to suicidal ideation and suicide attempts, ‘Context’ to studies conducted in Iran and ‘Population’ to university students.

### Search strategy for detection of relevant studies

We performed both manual and electronic searching and carried out a systematic search of several international databases, including PubMed, Web of Science, Scopus and PsycINFO, up to 3 September 2023. Following that, we updated the search up to 20 February 2025. Data were extracted starting on 5 November 2023, and updated data extraction began on 20 February 2025. We also used Magiran to find Persian papers. Our search strategy included the following keywords and Medical Subject Heading terms: Suicid* AND Iran* AND student*. Persian synonymous words were used to search the national database. Each database was searched with no restrictions on publication year or language. The full search syntaxes and number of records retrieved from each database are provided in the supplementary material available at https://doi.org/10.1192/bjo.2025.10921. We manually checked the reference lists of all included studies to identify any other eligible studies that may have been missed by our literature searches.

### Inclusion and exclusion criteria

The included studies were as follows: (a) all types of observational studies and (b) all epidemiological studies examining the occurrence of suicide attempts and suicidal ideation among Iranian university students. Studies with the following criteria were excluded: (a) animal studies, conference abstracts, comments, clinical trials, case reports, letters, any type of review, in vitro studies and case series; (b) duplicate publications; (c) qualitative investigations; (d) studies assessing the validity and reliability of questionnaires; and (e) studies that did not contain sufficient data to calculate the required parameters.

### Assessment of risk of bias (quality)

Two authors independently evaluated the quality of each study using the Newcastle–Ottawa Scale (NOS) checklist. NOS scoring ranges from 0 (lowest quality) to 10 (highest quality) and assesses studies in terms of group selection, group comparability and determination of the desired outcome. Considering that all studies were cross-sectional, we used the modified version of NOS.^23^ Because NOS has no standardised cut-offs, and arbitrary thresholds may vary across reviews, we reported individual NOS scores for each study descriptively rather than applying fixed categories. Disagreements were resolved through consensus and, if needed, were determined by another author.

### Screening and data extraction

One reviewer checked the titles and abstracts of the identified studies, then two reviewers independently assessed the full texts against the predefined eligibility criteria. Two other reviewers extracted the data independently; at each stage, differences between the two reviewers were resolved through consensus. If the disagreement between the authors was not resolved by consensus, another author as a third person made the final decision. The data extraction checklist involved the name of the first author, publication year, location of study, sample size, sampling method, mean age, percentage of suicide attempts and suicidal ideation.

### Data synthesis strategy

We analysed the data according to the guideline on conducting proportional meta-analyses across different types of systematic reviews,^24^ as well as the methodological guideline for performing meta-analysis of binomial data with Stata.^25^ We analysed the data using Stata/MP version 16.0 for Windows (StataCorp LLC, College Station, Texas, USA). In this research, we extracted the prevalence of suicidal ideation and suicide attempts with 95% confidence intervals; when prevalences were not reported in the original studies, we calculated these based on the available data. Heterogeneity among studies was calculated using the *I* ^2^ statistic and Cochran’s *Q*-test. Because we anticipated that the true prevalence could plausibly vary across studies (for example, due to differences in populations, settings and assessment instruments), we chose to use a random-effects model and pooled proportions with Stata’s metaprop command (Freeman–Tukey double-arcsine transformation).^25^ We also used meta-regression to explore potential sources of heterogeneity. We fitted random-effects, multivariable, meta-regression models, entering publication year, study quality (NOS score), total sample size and male:female ratio as predictors. After conducting meta-regression analyses, we produced bubble plots for each statistically significant moderator to visualise the relationship between effect size and moderator; these plots are provided in Supplementary Figs 1–3. To assess robustness, a leave-one-out sensitivity analysis was carried out, systematically removing one study at a time and recalculating the overall prevalence to evaluate the influence of each individual study on the final results.

## Results

### Literature search

**Fig. 1:**
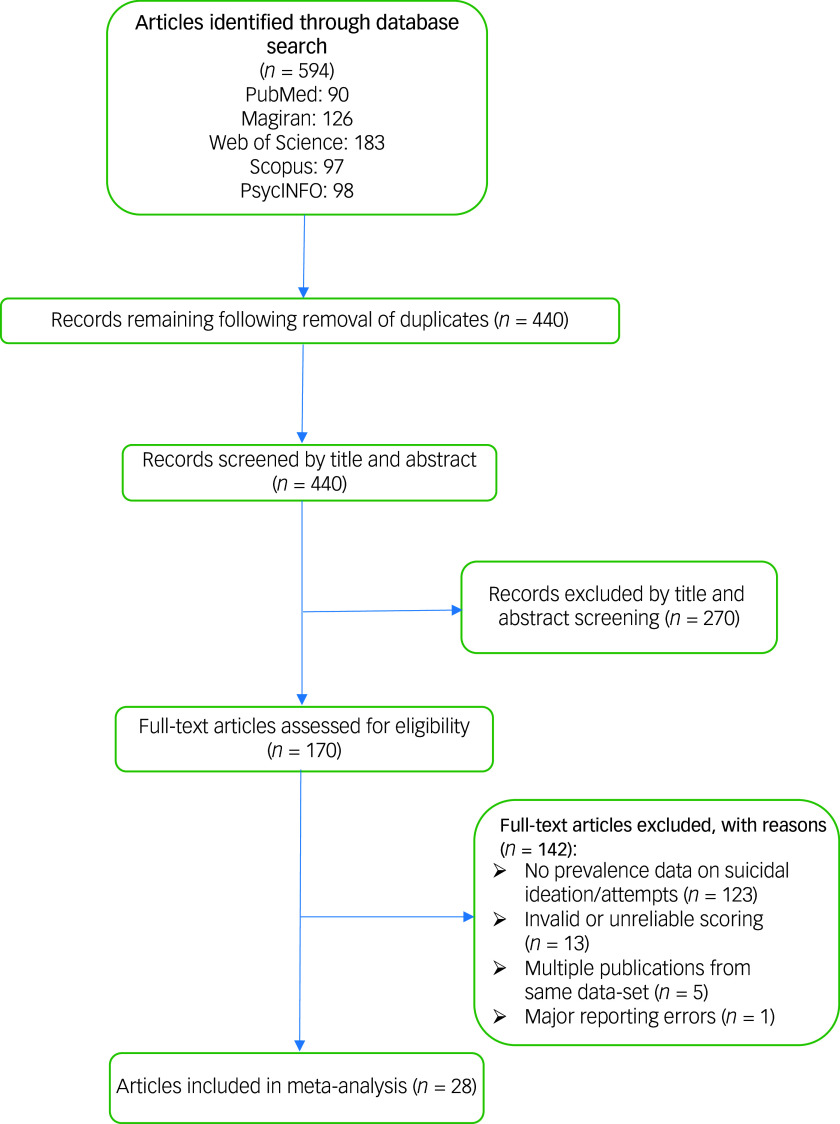
Preferred Reporting Items for Systematic Reviews and Meta-Analyses flowchart for the study selection process.

A total of 440 studies were identified after searching different databases and removing duplicates. A total of 170 articles were selected for full-text review. After the full texts were separately checked by the two reviewers, 141 studies were excluded because of not meeting the inclusion criteria. We also excluded one article due to substantial errors in reporting the results.^26^ The process of searching and eliminating those studies that did not satisfy the inclusion criteria is shown in Fig. 1.

### Study characteristics

Finally, 28 articles^4,14–18,27–48^ were included in the study. A total of 14 articles included information on suicide attempts^4,15,17,18,27–34,45,48^ and 17 included information regarding suicidal ideation.^14–16,34–47^ Among the 17 articles that assessed the prevalence of suicidal ideation, all but 3 used BSSI. In other words, according to our results, this instrument – despite its age – was the one most commonly used for assessment of suicidal ideation in Iran. Sample sizes ranged from 124 to 2181 participants. Sampling methods varied (e.g. cluster multi-stage random sampling, census and convenience sampling); however, some studies did not report their sampling method. Although we applied no restrictions on publication year, the earliest included study was published in 2013. The data extraction process is shown in Tables 1 and 2. In addition, studies were conducted in different cities of Iran.

**Table 1:** Data extraction file for prevalence of suicide ideation

Reference	Year of publication	University/ies	Type of study	Instrument	Sampling method	Total sample size	Sample size (men)	Sample size (women)	Mean age (years)	s.d. age	Total suicide ideation (%)	Men, suicide ideation (%)	Women, suicide ideation (%)
Saeed et al^45^	2024	Tehran University of Medical Science; Shahid Beheshti University of Medical Sciences; Iran University of Medical Sciences; AJA University of Medical Sciences; University of Social Welfare and Rehabilitation Sciences	Cross-sectional	Beck Scale for Suicidal Ideation (BSSI)	Convenience sampling	353	85	268	NR	NR	34.3	11.8	9.7
Ghorbanpour et al^46^	2024	Shiraz University of Medical Sciences	Cross-sectional	BSSI	Stratified random sampling	308	171	137	22.03	2.24	24.7	22.8	27
Mahdavi et al^47^	2023	School of Medical Sciences of Asadabad City	Cross-sectional	BSSI	Census	165	73	92	NR	NR	9.69	4.1	14.13
Nakhostin-Ansari et al^15^	2022	Tehran University of Medical Sciences	Cross-sectional	General Health Questionnaire (GHQ)	Convenience sampling	419	202	217	NR	NR	32	30.19	33.64
Sadeghian et al^35^	2021	Tehran University of Medical Sciences	Cross-sectional	GHQ	Available sampling	224	84	140	22.68	3.38	16.07	11.9	18.6
Habibi et al^36^	2021	Rafsanjan University of Medical Sciences	Cross-sectional	BSSI	Census	209	89	120	21.24	1.41	17.7	19.1	16.6
Ariapooran & Sheybani^37^	2021	Malayer University and Payam-e Noor University of Semnan	Cross-sectional	BSSI	Convenience sampling	311	124	187	22.89	3.12	14.47	11.29	16.58
Rohani & Esmaeili^16^	2020	University of Isfahan	Cross-sectional	Suicide Ideation Scale	Multi-stage sampling	180	0	180	22.36	4.75	25	NR	NR
Arezoo Heshmati et al^38^	2019	Zanjan University of Medical Sciences	Cross-sectional	BSSI	Convenience sampling	400	147	253	23.2	3.6	6	10.2	3.6
Ghaderi et al^39^	2018	Urmia Islamic Azad University	Cross-sectional	BSSI	Cluster multi-stage random sampling	396	NR	396	20.95	6.32	12	NR	12
Bashardoost & Ashori^40^	2016	Lahijan Islamic Azad University	Cross-sectional	BSSI	Random sampling	124	62	62	NR	NR	16.12	NR	NR
Niusheh Soofi et al^14^	2016	Rafsanjan University of Medical Sciences	Cross-sectional	BSSI	Census	265	126	139	NR	NR	6	7	5
Rahimi & Aradolahi^17^	2017	Rafsanjan University of Medical Sciences	Cross-sectional	BSSI	Census	233	86	147	21.97	3.67	14.4	NR	NR
Mirzaie & Shams Alizadeh^34^	2013	Kurdistan University of Medical Sciences	Cross-sectional	BSSI	Stratified random sampling	452	155	297	21.16	2.41	16.8	NR	NR
Mohammadinia et al^42^	2012	Iranshahr University of Medical Sciences	Cross-sectional	BSSI	Census	288	74	214	22.52	2.99	26.4	44.5	20
Mousavi et al^43^	2012	Three universities of Isfahan	Cross-sectional	BSSI	Convenience stratified sampling	435	181	254	21.76	2.33	7.58	NR	NR
Zemestani et al^44^	2023	Five universities of Kurdistan	Cross-sectional	Beck Suicide Scale (BSS-5)	Via university social media	679	228	451	24.34	4.86	16.5	NR	NR

NR, not reported.

**Table 2:** Data extraction file for prevalence of suicide attempts

Reference	Year of publication	University/ies	Type of study	Sampling method	Total sample size	Sample size (men)	Sample size (women)	Mean age (years)	s.d. age	Period of time	Total suicide attempts (%)	Men, suicide attempts (%)	Women, suicide attempts (%)
Yaghubi et al^4^	2024	Iranian University	Cross-sectional	Internet-based	2181	705	1476	NR	NR	Lifetime	7.5	NR	NR
Saeed et al^45^	2024	Tehran University of Medical Sciences; Shahid Beheshti University of Medical Sciences; Iran University of Medical Sciences; AJA University of Medical Sciences; University of Social Welfare and Rehabilitation Sciences	Cross-sectional	Convenience sampling	353	85	268	NR	NR	Lifetime	14.44	NR	NR
Ahmadboukani et al^48^	2022	University of Mohaghegh Ardabili	Cross-sectional	Convenience sampling	600	139	461	NR	NR	Lifetime	14.3	NR	NR
Nakhostin-Ansari et al^15^	2022	Tehran University of Medical Sciences	Cross-sectional	Convenience sampling	419	202	217	NR	NR	Lifetime	4.3	NR	NR
Narimani et al^18^	2020	Ardabil University of Medical Sciences	Cross-sectional	Stratified sampling	215	125	90	NR	NR	Past year	7.9	4.8	12.2
Ahmadpoor et al^27^	2020	13 medical science universities	Cross-sectional	NR	4261	1882	2379	22.17	3.18	Past year	1.7	NR	NR
Eskin et al^28^	2020	Iranian universities	Cross-sectional	NR	1000	400	600	22.43	3.93	Past year	4.0	NR	NR
Eskin et al^28^	2020	Iranian universities	Cross-sectional	NR	1000	400	600	22.43	3.93	Lifetime	5.0	NR	NR
Arayeshgari et al^29^	2020	Hamadan University of Medical Sciences	Cross-sectional	NR	1259	494	765	22.54	3.34	Lifetime	8.26	NR	NR
Arayeshgari et al^29^	2020	Hamadan University of Medical Sciences	Cross-sectional	NR	1259	494	765	22.54	3.34	Past month	3.9	NR	NR
Arayeshgari et al^29^	2020	Hamadan University of Medical Sciences	Cross-sectional	NR	1259	494	765	22.54	3.34	Past year	3.73	NR	NR
Poorolajal et al^17^	2018	13 medical science universities	Cross-sectional	NR	4261	1877	2379	22.17	3.18	Past year	1.7	NR	NR
Eskin et al^30^	2018	Iranian universities	Cross-Sectional	NR	700	350	350	22.1	4.0	Lifetime	5.1	NR	NR
Vasegh & Ardestani^31^	2018	Shahid Beheshti University of Medical Sciences	Cross-sectional	NR	421	136	285	20	2.0	Past year	3.8	NR	NR
Vasegh & Ardestani^31^	2018	Shahid Beheshti University of Medical Sciences	Cross-sectional	NR	421	136	285	20	2.0	Past month	0.5	NR	NR
Vasegh & Ardestani^31^	2018	Shahid Beheshti University of Medical Sciences	Cross-sectional	NR	421	136	285	20	2.0	Lifetime	7.8	NR	NR
Chalmardi et al^32^	2018	University of Mohaghegh Ardabili	Cross-sectional	Convenience sampling	600	NR	NR	NR	NR	Lifetime	12.0	NR	NR
Aliverdinia & Usefi^33^	2014	Mazandaran University	Cross-sectional	Stratified random sampling	438	149	289	NR	NR	Past year	2.5	2.0	2.8
Aliverdinia & Usefi^33^	2014	Mazandaran University	Cross-Sectional	Stratified random sampling	438	149	289	NR	NR	Lifetime	4.6	4.0	4.8
Mirzaie & Shams Alizadeh^34^	2013	Kurdistan University of Medical Sciences	Cross-sectional	Stratified random sampling	452	155	297	21.16	2.41	Lifetime	4.6	NR	NR

NR, not reported.

### Methodological quality

The methodological quality of the included studies varied; although some studies scored highly on NOS, several others had notable limitations. In particular, some studies did not receive a star in the statistical test domain because confidence intervals were not reported. A number of studies also provided no description of response rates, nor the characteristics of respondents versus non-respondents. Additionally, some studies did not stratify or adjust for key confounders such as gender or age, resulting in lower scores in the comparability domain. These issues may have affected the accuracy of prevalence estimates and contributed to heterogeneity. The quality ratings of each article are presented in full in the supplementary material.

### Prevalence of suicidal ideation and suicide attempts

**Fig. 2:**
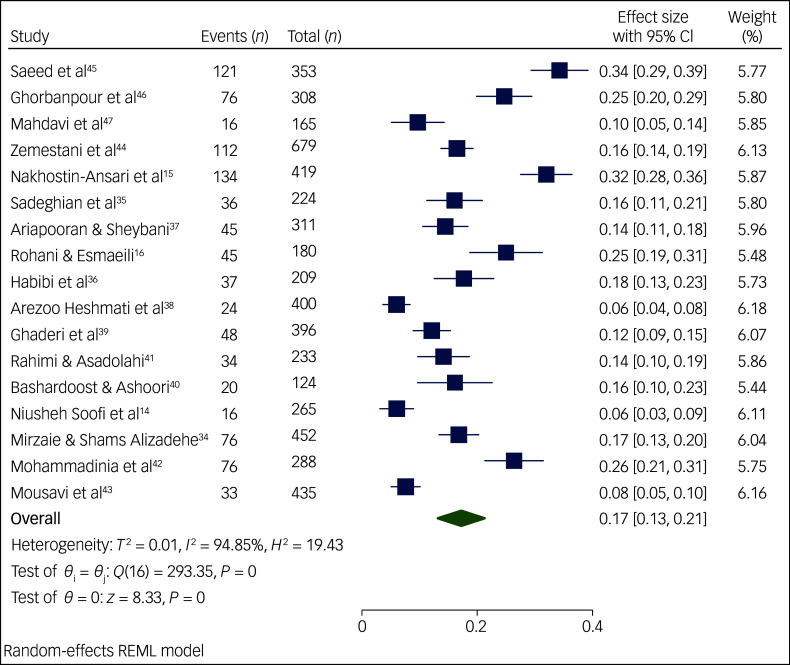
Prevalence of suicidal ideation among Iranian students. REML, restricted maximum likelihood.

**Fig. 3:**
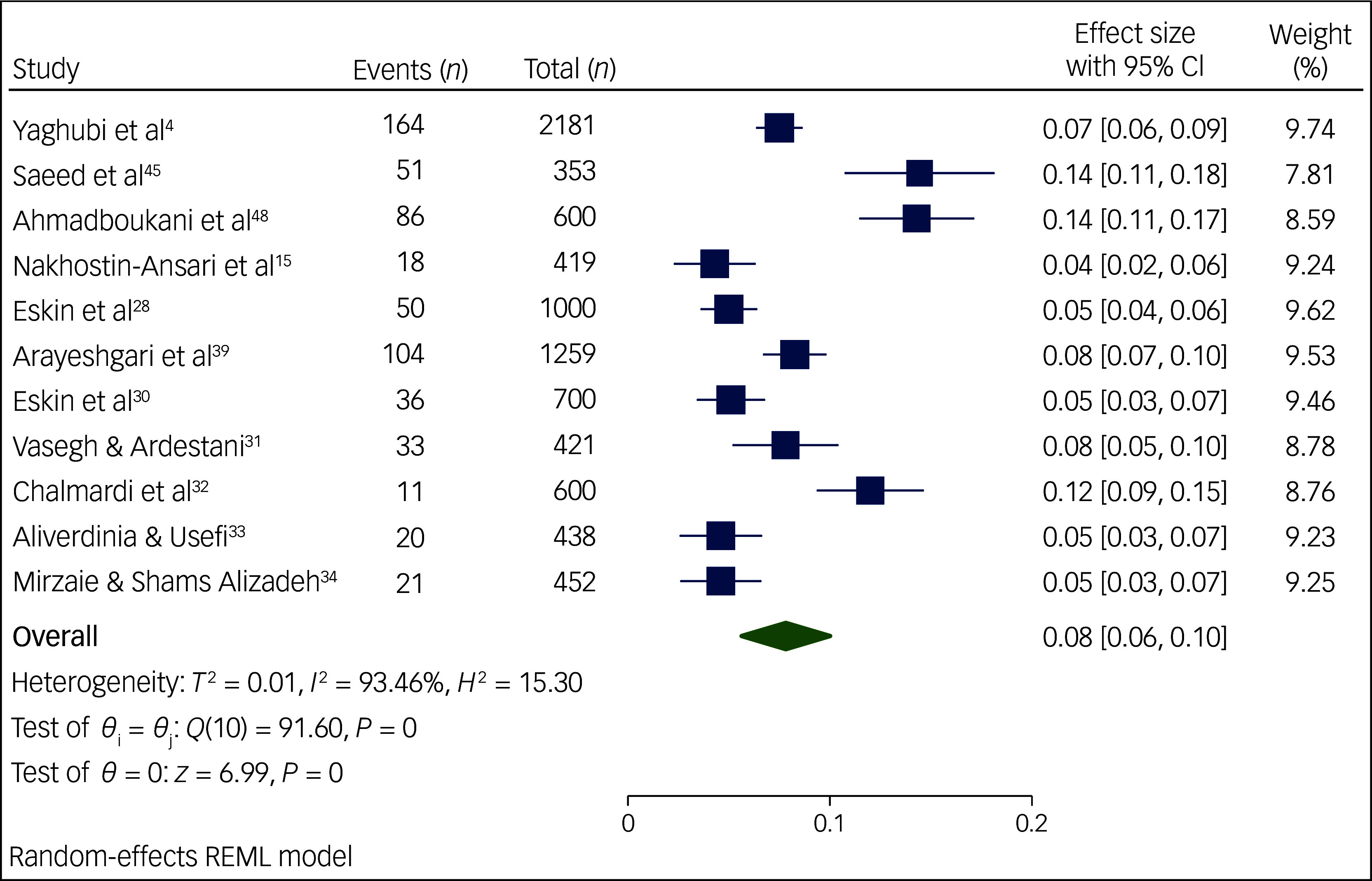
Prevalence of lifetime suicide attempts among Iranian students. REML, restricted maximum likelihood.

**Fig. 4:**
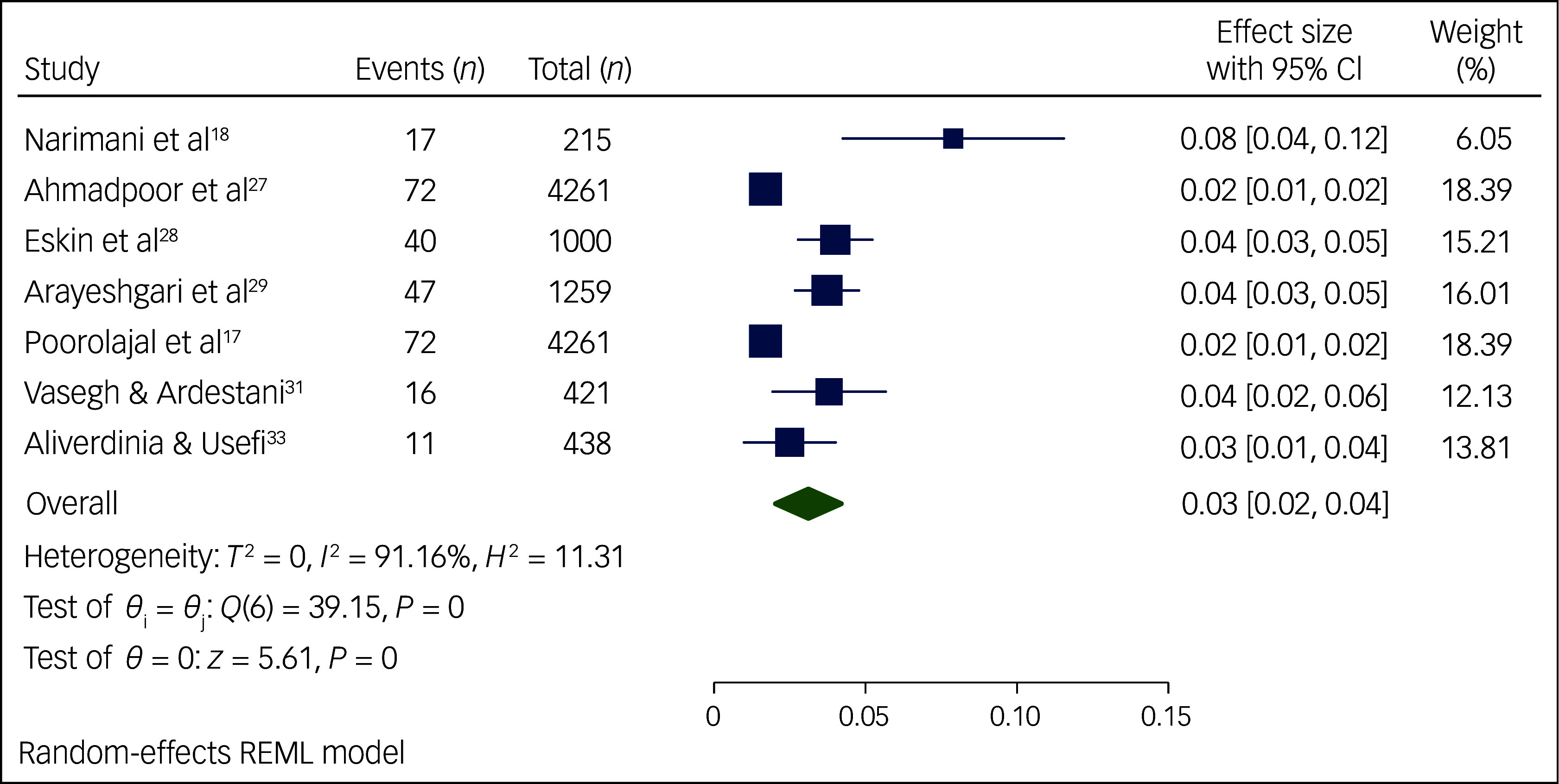
Prevalence of past-year suicide attempts among Iranian students. REML, restricted maximum likelihood.

The meta-analysis performed on the occurrence of suicidal ideation among Iranian students (Fig. 2) included 17 articles published between 2012 and 2024. Study heterogeneity was strong (*I* ^2^ = 94.85%, *P* < 0.01). Results from the forest plot indicated 17% pooled suicidal ideation among Iranian students, with a 95% CI of 13–21%. Similarly, the meta-analysis on suicide attempts among Iranian students (Figs 3 and 4) comprised 14 articles published between 2013 and 2024. We performed a meta-analysis of the prevalence of suicide attempts, both lifetime and for the past year, thereby obtaining the lifelong prevalence of suicide attempts as 8% with a 95% CI of 6–10%; we also obtained the pooled prevalence rate of suicide attempts in the past year at 3%, with a 95% CI of 2–4%. Again, study heterogeneity was strong for both lifelong prevalence (*I* ^2^ = 93.46%, *P* < 0.01) and past-year prevalence (*I* ^2^ = 91.16%, *P* < 0.01). Given the substantial heterogeneity in the meta-analyses of suicidal ideation and suicide attempts, we conducted sensitivity analyses and meta-regression, the outcomes of which will now be elaborated upon.

### Sensitivity analysis

**Fig. 5:**
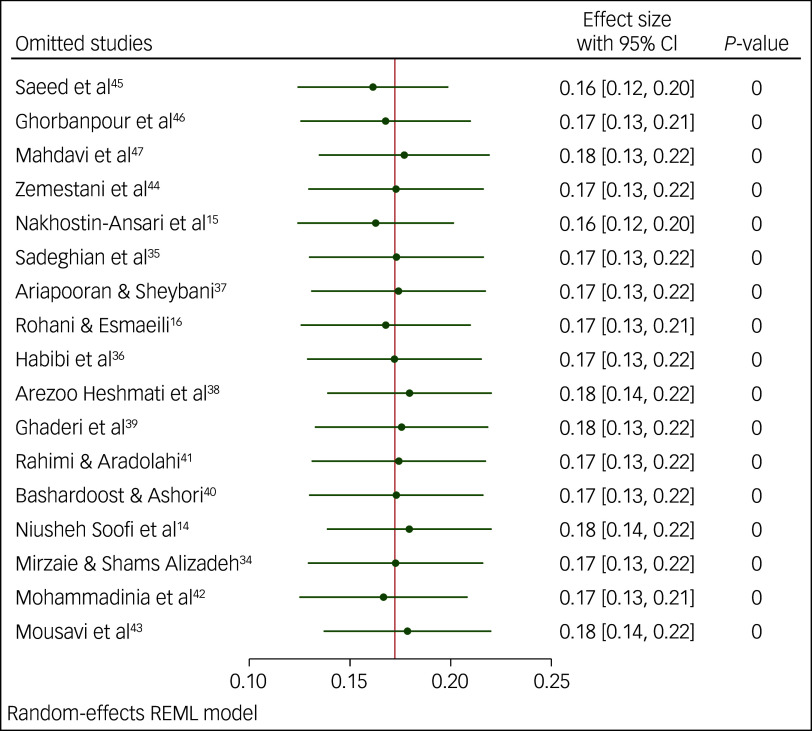
Sensitivity analysis for prevalence of suicidal ideation among Iranian students. REML, restricted maximum likelihood.

**Fig. 6:**
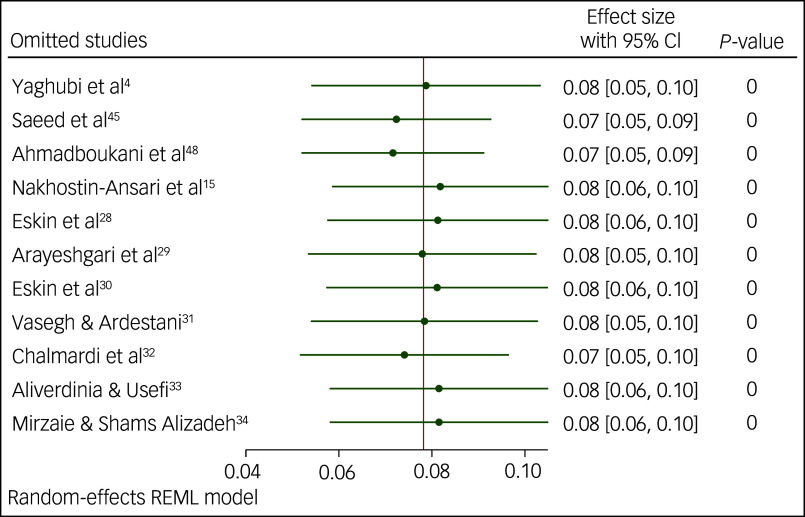
Sensitivity analysis for lifetime prevalence of suicide attempts among Iranian students. REML, restricted maximum likelihood.

**Fig. 7:**
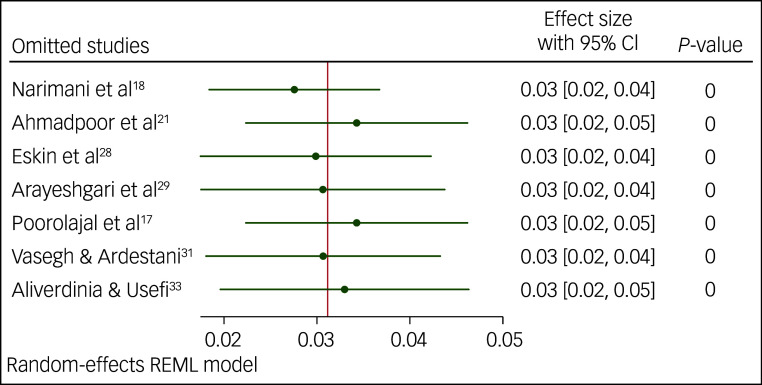
Sensitivity analysis for prevalence of past-year suicide attempts among Iranian students. REML, restricted maximum likelihood.

We performed a sensitivity analysis for the prevalence of suicidal ideation and suicide attempts using the leave-one-out method. The results of this analysis indicated that none of the point estimates for the occurrence of suicidal ideation (Fig. 5), or attempts of suicide (Figs 6 and 7), deviated from the overall 95% confidence interval. In essence, fluctuations in the prevalence of suicidal ideation ranged 16–18%, variations in the occurrence of lifetime attempts of suicide ranged 7–8% and the point estimation for past-year suicide attempt was 3%, all of which were statistically significant (*P* < 0.001).

### Results of meta-regression analysis

We used meta-regression to identify the sources of heterogeneity. In the suicidal ideation analysis, none of the examined moderators were significantly associated with effect size. In the lifetime suicide attempts analysis, publication year (*P* = 0.008) and male:female ratio (*P* = 0.002) were significantly associated with effect size (i.e. the prevalence of lifetime suicide attempts), so that more recent studies and a higher proportion of women were both associated with higher prevalence. In the analysis of past-year suicide attempts, only sample size showed a significant association with effect size (*P* < 0.001), with smaller studies reporting higher prevalence rates (Table 3).

**Table 3:** Meta-regression analyses on the prevalence of suicide attempts (lifetime, past year) and suicidal ideation

Parameters analysed	Coefficient	95% CI	s.e.	*P*-value
Suicide attempts (lifetime)				
Year^a^	0.006	(0.002, 0.010)	0.002	0.008
Quality assessment^b^	−0.005	(−0.018, 0.008)	0.006	0.425
Sample size^c^	0.000	(−0.00004, 0.000016)	0	0.344
Male/female^d^	−0.100	(−0.162, −0.038)	0.032	0.002
Suicide attempts (past year)				
Year	0.001	(−0.001, 0.003)	0.001	0.296
Quality assessment	0.001	(−0.002, 0.006)	0.002	0.523
Sample size	−7.92 × 10^−6^	(−0.000011, −4.890 × 10^−6^)	1.55 × 10^−6^	<0.001
Male/female	0.043	(−0.002, 0.890)	0.023	0.061
Suicidal ideation				
Year	0.008	(−0.001, 0.019)	0.005	0.102
Quality assessment	0.024	(−0.010, 0.059)	0.177	0.170
Sample size	0.000	(−0.0003, 0.0002)	0	0.711
Male/female	−0.035	(−0.164, 0.093)	0.065	0.588

a. Publication year. b. Newcastle–Ottawa quality score (higher score denotes better quality). c. Total participants (units, 1). d. Male:female ratio.

## Discussion

The aim of this study was to estimate the prevalence rates of suicidal ideation and suicide attempts among university students in Iran. According to our meta-analysis, the pooled prevalence of suicidal ideation and 12-month and lifetime suicide attempts among Iranian students was 17% (95% CI: 13–21%), 3% (95% CI: 2–4%) and 8% (95% CI: 6–10%), respectively.

By conducting a meta-analysis of 17 studies, we found that the current prevalence of suicidal ideation in Iranian students is 17% (95% CI: 13–21%). Based on a systematic review and meta-analysis,^49^ the current and 12-month prevalence of suicidal ideation in students of Muslim-majority countries is 6.4% (95% CI: 4.5–9%) and 13.4% (95% CI: 11.1–16.1%), respectively. In addition, Mortier et al^10^ found that the worldwide 12-month frequency of suicidal ideation in college students was 10.6% (95% CI: 9.1–12.3%). It appears that students of Muslim-majority countries, including Iran, exhibit more frequent suicidal ideation than other students around the world.^10,49^ The higher suicidal ideation of students in Muslim-majority countries is probably due to their harsh living conditions. Extensive economic sanctions, high inflation, shortage of medicines and medical equipment, administrative corruption, lack of democracy and the non-existence of security are among the problems inherent in these countries.^50^ These difficulties can have negative effects on students’ mental health in the long term. Despite these conditions, religion as a source of meaning in life can probably act as an defence against suicide in Muslim-majority countries.^51–53^ Moreover, Islamic teachings consider suicide a greater sin (*kabīrah*), and this strong religious prohibition could reduce suicide rates in Muslim societies.^54^

In this research, by performing a meta-analysis on 7 studies, we found that the 12-month prevalence of suicide attempts in Iranian students is 3% (95% CI: 2–4%); and, using a meta-analysis of 11 studies, we found that the lifetime prevalence of suicide attempts in Iranian students is 8% (95% CI: 6–10%). According to another study, the occurrence of suicide attempts in the general population of Iran was 131 per 100 000 people,^55^ which is clearly lower than the rate among students. This variation in the prevalence of suicide attempts between students and the general population is probably due to the more difficult living conditions of the former. If a person does not have a valued meaning in life, problematic living conditions and mental pressures can lead to suicide, with the aim of relieving pain.^56^ The results of a research conducted in 2022 in one of Iran’s universities also showed that about 95% of students felt they had no meaning in life and that they were not searching for any.^2^ According to one meta-analysis,^10^ the frequency of suicide attempts in college students worldwide during their lifetime and over the past year was 3.2% (95% CI: 2.2–4.5%) and 1.2% (95% CI: 0.8–1.6%), respectively, which is lower than the suicide attempt rate for Iranian students. According to another meta-analysis,^49^ suicide attempts among students of Muslim-majority countries during their lifetime and over the past year were 6.6% (95% CI: 5.4–8%) and 4.9% (95% CI: 3.6–6.5%), respectively.

Considering the relatively high prevalence of suicidal ideation and attempts among Iranian university students, measures should be taken as soon as possible to resolve this problem. Furthermore, there are limited suicide-related studies in Iran, and these investigations usually have methodological flaws. The government should continuously monitor the prevalence of suicidal ideation and behaviour among students, with extensive and long-term planning, in order to find both protective and risk factors in each subculture separately. Efforts to destigmatise mental disorders and psychologist consultation,^50^ continuous logotherapy of students^57^ and increasing the amount of education loans to solve economic problems are probably appropriate short-term solutions.

The high level of heterogeneity observed in our meta-analysis is a common challenge in prevalence studies,^58^ reflecting the diverse methodologies, populations and measurement tools used in the included studies. Meta-regression showed that, for lifetime suicide attempts, publication year was positively associated with the pooled prevalence, and that the male:female ratio was negatively associated with it; for past-year suicide attempts, sample size was negatively associated with the estimates (consistent with small-study effects). Nevertheless, residual heterogeneity remained substantial and, with the available moderators, we could not fully account for between-study variability. This unresolved heterogeneity underscores the need for future studies with standardised methodologies and consistent reporting of key variables (e.g. demographic characteristics and study design features). Furthermore, exploring broader contextual contributors – such as cultural, regional or institutional differences – may provide greater insight into factors driving the variability in suicidal ideation and attempts among Iranian university students.

### Limitations

The study we conducted has a number of limitations, most of which we encountered stem from those of the primary studies. First, the majority of studies used BSSI^59^ to measure the prevalence of suicidal ideation. Some studies had scored the scale in new ways, but none reported evidence for the validity and reliability of their new scoring methods. In the present research, to measure the occurrence of suicidal ideation we included only those studies that used valid scales and scoring methods. Second, due to the nature of this work (a prevalence meta-analysis), heterogeneity was high and its sources could not be fully identified. Third, the studies we reviewed estimated prevalence at different times and in various locations; given the scarcity of studies, we could not determine whether suicide has decreased or increased over time. Another limitation was the small number of studies conducted within Iran’s various subcultures. Iran comprises multiple subcultures and, in this study, we were unable to estimate suicide prevalence separately for each subculture. We also found that several questionnaires had been used across studies to measure suicidal ideation; this may be one of the reasons for differences in prevalence rates between countries. It is not possible to specify the direction of this effect – that is, whether the use of different questionnaires tends to increase or decrease reported prevalence. Finally, although the protocol for this review was registered in PROSPERO (no. CRD42023471340), the statistical analysis section in the registered record was mistakenly written based on a different, unrelated study. Nevertheless, the analyses actually conducted in this review were appropriately designed in accordance with its specific objectives and data-set.

### Clinical implications

The findings of this study indicate that suicidal ideation and suicide attempts are highly prevalent among Iranian university students. This has serious implications for both public health and higher education, and universities should consider suicidality as a major priority in student health policies. Early detection and prevention programmes must be systematically implemented in universities. Counselling centres should be strengthened and made more accessible. Awareness campaigns and peer-support initiatives are also needed to reduce the stigma around mental health services. Routine screening for suicidal ideation could help identify students at risk before a crisis occurs.

### Future directions

This systematic review and meta-analysis highlights the prevalence of suicidal ideation and suicide attempts among Iranian university students, emphasising the urgent need for more robust and comprehensive research in this area. The current body of research in Iran is not only limited, but also marked, by significant methodological and statistical shortcomings, including small and unrepresentative samples, lack of standardised tools for assessment of suicidal behaviours and inconsistent data reporting.

Future studies should focus on overcoming these limitations through employing rigorous methodologies, such as larger, more diverse and representative samples, standardised instruments for measuring suicidal ideation and suicide attempts, and advanced statistical analyses. Moreover, there is a pressing need for longitudinal studies to explore causal relationships and trends over time, as well as mixed-methods research to provide deeper insights into the underlying cultural, social and academic factors that contribute to suicidal behaviours.

Efforts should also be directed towards developing and evaluating culturally appropriate interventions, such as mental health education programmes, accessible counselling services and peer support networks, to reduce the prevalence of suicidal ideation and suicide attempts in this population. Moreover, fostering collaboration among universities, mental health organisations and policy-makers will be critical to designing and implementing effective, evidence-based strategies for suicide prevention among university students in Iran.

## Supporting information

Mahdavinoor et al. supplementary material 1 Mahdavinoor et al. supplementary material

Mahdavinoor et al. supplementary material 2 Mahdavinoor et al. supplementary material

Mahdavinoor et al. supplementary material 3 Mahdavinoor et al. supplementary material

## Data Availability

Following reasonable request, the data will be made available by the corresponding author.

## Supplementary material

The supplementary material is available online at https://doi.org/10.1192/bjo.2025.10921
