# Supplementary material for: Prevalence of suicidal ideation and suicide attempts among Iranian university students: systematic review and meta-analysis
Source: BJPsych Open. 2026 Jan 6;12(1):e27. doi: 10.1192/bjo.2025.10921 (PMC12835722; doi:10.1192/bjo.2025.10921)
Supplement: Mahdavinoor et al. supplementary material 1 — Mahdavinoor et al. supplementary material [file S2056472425109216sup001.docx]

Figure S1: Bubble Plot of 12-Month Prevalence of Suicide Attempts vs. Sample Size

Figure S2: Bubble Plot of Lifetime Prevalence of Suicide Attempts vs. Male-to-Female Ratio

Figure S3: Bubble Plot of Lifetime Prevalence of Suicide Attempts vs. Publication Year
