# Supplementary material for: Prevalence of suicidal ideation and suicide attempts among Iranian university students: systematic review and meta-analysis
Source: BJPsych Open. 2026 Jan 6;12(1):e27. doi: 10.1192/bjo.2025.10921 (PMC12835722; doi:10.1192/bjo.2025.10921)
Supplement: Mahdavinoor et al. supplementary material 2 — Mahdavinoor et al. supplementary material [file S2056472425109216sup002.docx]

**Newcastle-Ottawa Scale Quality Assessment Results**

**(Adapted for cross sectional studies)**

| Domain and topic | | | | | | | | | | | | | | | | | | | | | | | Row |
| --- | --- | --- | --- | --- | --- | --- | --- | --- | --- | --- | --- | --- | --- | --- | --- | --- | --- | --- | --- | --- | --- | --- | --- |
| Total score and study quality | Outcome 2 | | Outcome 1 | | | | Comparability | | Selection 4 | | | Selection 3 | | | Selection 2 | | Selection 1 | | | | Year | Author |  |
|  | not appropriate, not described or incomplete Statistical test | Clearly described and appropriate of Statistical test | No description | Self-report | Record linkage | Independent blind assessment | additional factor | most important factor | No description | Non-validated measurement tool | Validated measurement tool | No description | The response rate is unsatisfactory | Established comparability between respondents and non-respondent | Not justified | Justified and satisfactory | No description of the sampling strategy | Selected group of users | Somewhat representativeness of the average in the target population | Representativeness of the average in the target population |  |  |  |
| 8 |  | * |  |  |  | ** | * | * |  |  | ** | - |  |  | - |  |  |  | * |  | 2022 | A. Nakhostin-Ansari | 1 |
| 9 |  | * |  |  |  | ** | * | * |  |  | ** | - |  |  |  | * |  |  | * |  | 2021 | M.H Sadeghian | 2 |
| 8 |  | * |  |  |  | ** |  | * |  |  | ** |  |  | * |  |  |  |  |  | * | 2020 | S. Narimani | 3 |
| 8 |  | * |  |  |  | ** | * | * |  |  | ** | - |  |  | - |  |  |  |  | * | 2021 | F. Habibi | 4 |
| 7 |  | * |  |  |  | ** |  | * |  |  | ** |  | - |  |  |  |  |  | * |  | 2020 | J. Ahmadpoor | 5 |
| 6 |  | * |  |  |  | ** |  |  |  |  | ** | - |  |  | - |  |  |  | * |  | 2021 | S. Ariapooran | 6 |
| 6 |  | * |  |  |  | ** |  |  |  |  | ** | - |  |  | - |  |  |  |  | * | 2020 | F. Rohani | 7 |
| 8 |  | * |  |  |  | ** |  | * |  |  | ** | - |  |  |  | * |  |  | * |  | 2020 | M. Eskin | 8 |
| 9 |  | * |  |  |  | ** |  | * |  |  | ** | - |  | * |  | * |  |  |  | * | 2020 | M. Arayeshgari | 9 |
| 8 |  | * |  |  |  | ** | * | * |  |  | ** | - |  |  | - |  |  |  |  | * | 2018 | J. Poorolajal | 10 |
| 6 |  | * |  | * |  |  | * | * |  | * |  | - |  |  | - |  |  |  | * |  | 2018 | M. Eskin | 11 |
| 6 | - |  |  |  |  | ** |  |  |  |  | ** | - |  |  |  | * |  |  |  | * | 2019 | A. Heshmati Joda | 12 |
| 6 |  | * |  |  |  | ** |  | * |  | * |  | - |  |  | - |  |  |  |  | * | 2018 | S. Vasegh | 13 |
| 8 | - |  |  |  |  | ** |  | * |  |  | ** |  |  | * |  | * |  |  |  | * | 2018 | D. Ghaderi | 14 |
| 5 | - |  |  |  |  | ** |  |  |  |  | ** | - |  |  | - |  |  |  | * |  | 2018 | A. Kiani Chalmardi | 15 |
| 5 | - |  |  |  |  | ** |  |  |  |  | ** | - |  |  | - |  |  |  |  | * | 2016 | B. Bashardoost | 16 |
| 6 |  |  |  |  |  | ** |  |  |  |  | ** | - |  |  |  | * |  |  |  | * | 2016 | N. Soofi Afshar | 17 |
| 7 |  | * |  |  |  | ** |  |  |  |  | ** | - |  |  |  | * |  |  |  | * | 2017 | N. Rahimi | 18 |
| 7 |  | * |  |  |  | ** |  |  |  |  | ** | - |  |  |  | * |  |  |  | * | 2014 | A. Aliverdinia | 19 |
| 9 |  | * |  |  |  | ** | * | * |  |  | ** | - |  |  |  | * |  |  |  | * | 2013 | S. N. Mirzaie | 20 |
| 7 |  | * |  |  |  | ** |  |  |  |  | ** |  |  |  |  | * |  |  |  | * | 2012 | N. Mohammadinia | 21 |
| 8 |  | * |  |  |  | ** |  | * |  |  | ** | - |  |  |  | * |  |  |  | * | 2012 | S.G. Mousavi | 22 |
| 6 |  | * |  |  |  | ** |  |  |  |  | ** | - |  |  | - |  |  |  |  | * | 2023 | M. Zemestani | 23 |
| 9 |  | * |  |  |  | ** | * | * |  |  | ** |  | - |  |  | * |  |  | * |  | 2024 | H. Yaghubi | 24 |
| 8 | - |  |  |  |  | ** | * | * |  |  | ** |  | - |  |  | * |  |  | * |  | 2024 | F. Saeed | 25 |
| 8 |  | * |  |  |  | ** |  | * |  |  | ** | - |  |  |  | * |  |  |  | * | 2024 | A. Ghorbanpour | 26 |
| 6 | - |  |  |  |  | ** |  |  |  |  | ** |  | - |  |  | * |  |  |  | * | 2023 | A. Mahdavi | 27 |
| 6 | - |  |  |  |  | ** | * | * |  |  | ** | - |  |  | - |  | - |  |  |  | 2022 | S. Ahmadboukani | 28 |
