# Supplementary material for: Prevalence of suicidal ideation and suicide attempts among Iranian university students: systematic review and meta-analysis
Source: BJPsych Open. 2026 Jan 6;12(1):e27. doi: 10.1192/bjo.2025.10921 (PMC12835722; doi:10.1192/bjo.2025.10921)
Supplement: Mahdavinoor et al. supplementary material 3 — Mahdavinoor et al. supplementary material [file S2056472425109216sup003.docx]

| **Database** | **Date searched** | **Syntax** | **Initial (N)** | **Update (N)** | **Total (N)** |
| --- | --- | --- | --- | --- | --- |
| PubMed | Sep 2023 / Feb 2025 | (Suicid*[Title/Abstract]) OR Suicide[MeSH Terms]) AND (Student*[Title/Abstract]) AND Iran | 71 | 19 | 90 |
| Scopus | Sep 2023 / Feb 2025 | ( TITLE-ABS-KEY ( suicid* ) AND TITLE-ABS-KEY ( iran* ) AND TITLE-ABS-KEY ( student* ) ) | 76 | 21 | 97 |
| Web of Science | Sep 2023 / Feb 2025 | TS=(Suicid*) AND ALL=(Iran) AND TS=(Student*) | 120 | 63 | 183 |
| MagIran | Sep 2023 / Feb 2025 | خودکشی و دانشجو | 111 | 15 | 126 |
| PsycINFO | Sep 2023 / Feb 2025 | TX Suicid* AND TX Iran AND TX Student* | 72 | 26 | 98 |
